# Epidemiology and burden of dengue in Cambodia

**Published:** 2011-01-10

**Authors:** Sirenda Vong, Rekol Huy, Sivuth Ong, Magali Teurlai, Sowath Ly, Julien Beauté, Veasna Duong, Philippe Buchy

**Affiliations:** 1 Epidemiology and Public Health Unit, Institut Pasteur in Cambodia, Phnom Penh, Cambodia; 2 National Center for Malariology, National Dengue Surveillance Program, Phnom Penh, Cambodia; 3 Virology Unit, Institut Pasteur in Cambodia, Phnom Penh, Cambodia

## Background

Dengue is endemic in Cambodia, affecting mainly children. We summarized our results of recent studies aiming to better understand the epidemiology of dengue and estimate its burden in Cambodia. We analyzed national surveillance data from 2002 thru 2008 to characterize temporal trends and space-time patterns of the disease. We conducted active community based surveillance of dengue during 2006-2010 among 0-19 year-olds to estimate dengue burden and understand the transmission dynamics of dengue in rural and urban areas in the largest province of Cambodia (census ~ 8,000 persons under surveillance each year). Comparing the two surveillance systems using capture-recapture methods we estimated the degree of under-recognition of dengue cases by national surveillance. We finally estimated economic burden of dengue using true incidence estimates and data regarding costs of illness among hospitalized and ambulatory cases.

## Results

During 2006 – 2008, overall incidence rates ranged from 1.3% - 5.7% among the 0-19 year-olds. Dengue is highly focal in its geographic distribution; incidence rates by village and year ranged widely from 0 – 13% in 2006 (one of 16 villages accounted for 50% of the cases), 1 – 22% in 2007-2008 (32% of villages had incidence rates >10%). More importantly, dengue affected rural areas to the same degree as urban areas or even higher in 2007 (7% vs. 1.7% in 2007 and 1.7% vs. 2.1% in 2006). The highest age-specific incidences lied among pre-school children (<8 year-olds). The degree of under-recognition from the national surveillance system varied widely from 4 to 29 fold from year to year. Exposure to flaviviruses was high at early ages: 85% of 3-4 year-old children had antibodies to flaviviruses in 2007 which may reflect intense transmission around the households. By using wavelet analysis to study the temporal lag between epidemics at different districts we show the existence of a recurrent annual pattern. The annual epidemic started in a limited number of rural districts. The propagation to the rest of the country from those few "sparks" three patterns of travelling waves along the 2 main roads and the Mekong River. The societal costs of dengue were high: US$4 – 16 million annually. Moreover, families support the highest share with ~78% of total costs and 67% incurred debts to pay for these costs.

## Conclusion

Costs of disease based on national surveillance data are not accurate. Our findings will become important factors in the public-health decision making process for Cambodia particularly the burden among the poor when balancing the benefits of introducing a potentially safe and effective dengue vaccine.

